# Changes in the healthcare utilization after establishment of emergency centre in Yaoundé, Cameroon: A before and after cross-sectional survey analysis

**DOI:** 10.1371/journal.pone.0211777

**Published:** 2019-02-08

**Authors:** So Yeon Joyce Kong, Dae Han Wi, Young Sun Ro, Sang Do Shin, Joongsik Jeong, Yun Jeong Kim, Joseph Kamgno, Etoundi Mballa Georges Alain, Bonaventure Hollong, Young Jin Oh

**Affiliations:** 1 Laboratory of Emergency Medical Services, Seoul National University Hospital Biomedical Research Institute, Seoul, Korea; 2 Department of Emergency Medicine, Seoul National University Hospital, Seoul, Korea; 3 Department of Emergency Medicine, Wonkwang University School of Medicine and Wonkwang University Sanbon Hospital, Gyeonggi, Korea; 4 Korea International Cooperation Agency, Cameroon Office, Yaoundé, Republic of Cameroon; 5 Yaoundé Emergency Center (Centre des Urgences de Yaoundé), Yaoundé, Cameroon; 6 Department of Emergency Medicine, Kyungpook National University Chilkok Hospital, Daegu, Korea; 7 Department of Public Health, Faculty of Medicine and Biomedical Sciences, University of Yaoundé I, Yaoundé, Republic of Cameroon; 8 Disease Control, Epidemics and Pandemics, Ministry of Public Health, Republic of Cameroon; University of Colorado, UNITED STATES

## Abstract

In effort to address the shortage of emergency medical care in Cameroon, the Yaoundé Emergency Center (CURY) was established in June, 2015 in Yaoundé, Cameroon. To evaluate its impact on the communities of Yaoundé, we assessed the changes in utilizations of emergency medical care since the establishment of the CURY. In 2014 the first survey was conducted on randomly selected 619 households (3,201 individuals) living in six health districts of Yaoundé. In 2017 the second quantitative survey was conducted on 622 households (3,472 individuals) using the same survey methods as the first survey. In both surveys, data on demographic information, socioeconomic status, and utilization of healthcare, including emergency care in the past year were collected on every member of the households via face-to-face interview. Data on two surveys were compared. Participants in the both surveys had similar age and gender distribution with mean age of 21–22 and 46% being male. In 2014 survey, healthcare utilization rates for emergency unit, outpatient, and hospitalization were 4.8%, 36.7%, and 10.0%, respectively. In 2017 survey, corresponding rates were 5.8%, 32.5%, and 9.2%%, respectively. The increase in the utilization of emergency unit between two surveys showed a marginal statistical significance (p = 0.08), while outpatient utilization showed statistically significant decrease from 2014 to 2017 survey (from 36.7% to 32.5%; p <0.001). After the establishment of a dedicated emergency medical center in Yaoundé, Cameroon, the utilization of emergency care was increased in the Yaoundé community. Further studies are warranted to examine the direct effect of the establishment of the CURY on healthcare utilization in Yaoundé.

## Introduction

The communities of sub-Saharan Africa face a disproportionate burden of acute illness and injury [1]. The rapidly growing prevalence and complications from non-communicable diseases [2, 3], such as cardiovascular and diabetes, further contribute to the need for appropriate emergency care services in the region. Despite its important and increasing role, however, the development of emergency care delivery system has largely been overlooked and neglected in these low-and middle-income countries (LMIC) in Africa [4]. As a result, to place emergency care on the global agenda, the World Health Assembly has called for all its member states to develop “formal, integrated emergency care systems’ by passing the resolution 60.22 in 2007 [5].

In effort to address the shortage of emergency care services, Korea International Cooperation Agency (KOICA), in collaboration with Cameroon Ministry of Public Health (MINSANTE), established the Yaoundé Emergency Center (CURY) in Yaoundé, Cameroon in June, 2015. In 2014, before the establishment of CURY, we conducted a community-based cross-sectional study to assess the burden of emergency illness and the utilization of healthcare, including emergency care by Yaoundé residents [6].

The establishment of this new emergency center is believed to increase the utilization of emergency care services in Yaoundé area, yet its impact on the community has not been evaluated. The objectives of this study are to assess the utilization of emergency care services by Yaoundé residents after the establishment of the CURY, and to evaluate the changes in utilization. We hypothesize that after the establishment of the CURY, there were increased utilization of emergency care services by the residents of Yaoundé.

## Materials and methods

### Ethical statements

Administrative authorization was obtained from the Regional Delegate of Yaoundé and Division of Operation Research in Health at the Cameroon Ministry of Public Health (No. 631–0217). This study was approved by the Seoul National University Hospital Institutional Review Board (No. H-1408-015-600) and the ethical clearance from the Ministry of Public Health of Cameroon (No. 0185/A/MINSANTE/SG/DRSPC).

### Study design

This is a second community-based cross-sectional study performed in Yaoundé, Cameroon. In December 2014, the first survey was conducted on randomly selected 619 households (3,358 individuals). Detailed information about study design, analysis, results, and conclusion of the study were previously published [6].

### Study setting

The Republic of Cameroon is a developing country located in Central Africa and has an estimated population of 23.4 million with life expectancy of 56 years and median age of 18. Gross national income per capita was estimated around 1,200 USD using the Atlas method in 2016 [7]. According to the World Health Organization (WHO) Statistics 2015, total health expenditures were 4.4% of gross domestic product (GDP) in Cameroon. Age-standardized mortality rates by communicable, non-communicable and injuries were 769, 675, and 106 per 100,000 population, respectively. Maternal mortality ratio was 596 per 100,000 live births [8].

**Fig 1:**
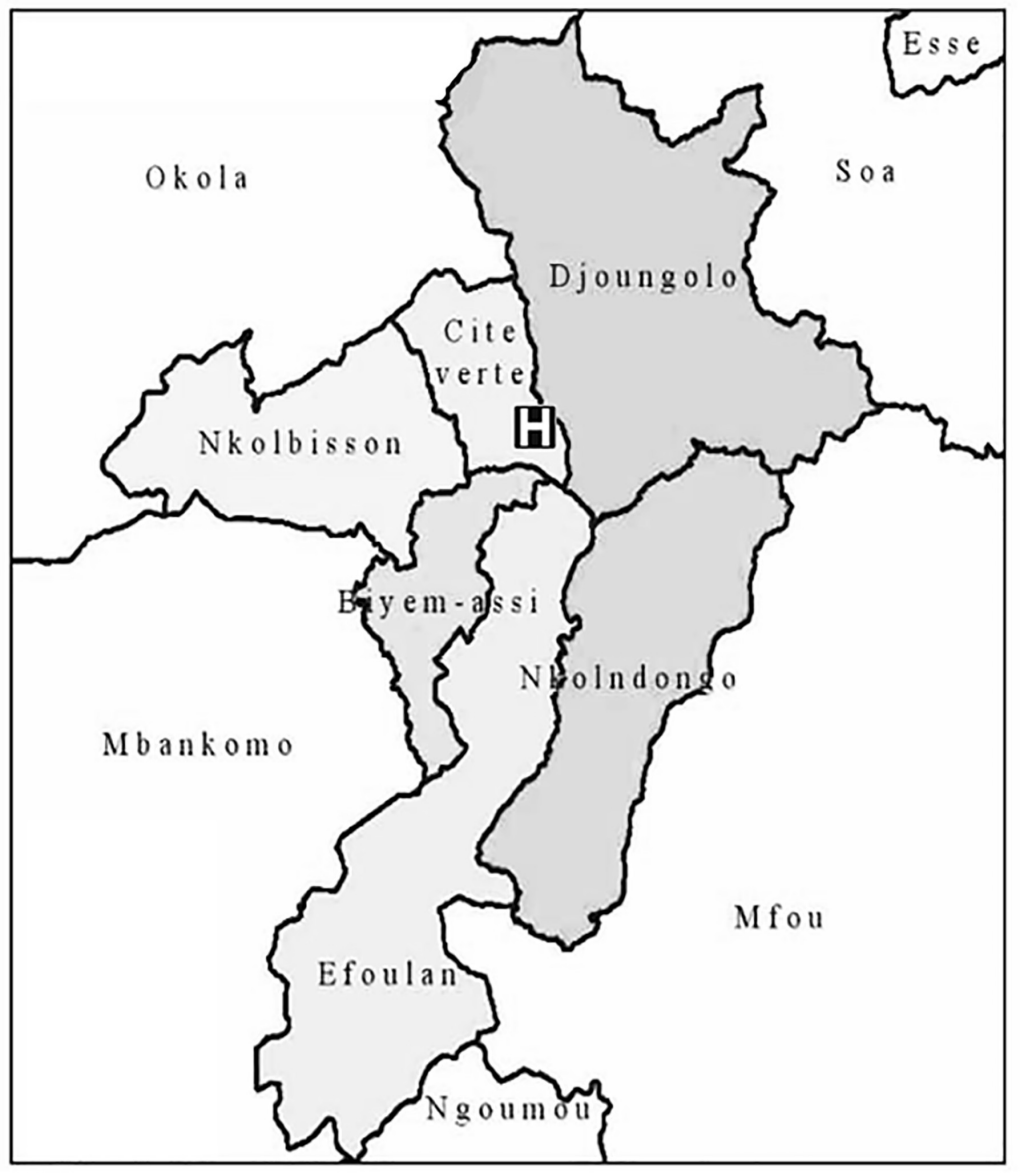
Six health districts of Yaoundé, Cameroon (Biyem assi, Cite verte, Djoungolo, Efoulan, Nkolbisson, and Nkolndongo). Centre des Urgences de Yaoundé (CURY), marked as “**H**” in the figure, is located in “Cite verte” health district. Reprinted from https://www.dhis-minsante-cm.org/portal/ under a CC BY license, with permission from Directorate of Disease Control, Epidemics, and Pandemics. Ministry of Public Health, Cameroon.

The capital city of Cameroon, Yaoundé with a population of approximately 3.2 million, is the second largest city in the country. The healthcare system in Cameroon is characterized by the division of the country into health districts, which is the operational geographic unit responsible for providing primary healthcare to the population [9]. There are six health districts (Biyem Assi, Cité Verte, Djoungolo, Efoulan, Nkolbisson, and Nkoldongo) in Yaoundé with each health district providing coverage for between 4 and 12 health areas and their constituting communities (Fig 1) [10]. Before CURY was established, there were a total of five tertiary care centres with four having emergency units and three receiving trauma patients [11].

Cameroon’s healthcare payment system is based on the fee-for-service model with poorly structured health insurance program. It was estimated that between 2010 and 2014, 94.2% of total private expenditure on health was out-of-pocket expenditure [7]. The WHO estimates the density of physicians as 0.8 and nurses and midwives as 5.2 per 10,000 population, which is far lower than the global average of 15.4 doctors and 32.7 nurses and midwives per 10,000 population, respectively [12, 13].

### Study population

All residents of Yaoundé were the target population for the study. Study population included those who lived in Yaoundé at the time of this study and agreed to participate. Among those who agreed to participate in the survey, one participant older than 21 year of age were selected in each sampled household. The households that had no adult older than 21 at the time of the visit were excluded from the study. Incomplete household data and those with unknown information on age and gender were also excluded in the analysis.

### Sample size and sampling method

Sample size was extrapolated using the WHO/EPI (Expanded Program on Immunization) cluster sampling method, which was established and initiated in 1974 by WHO to rapidly assess vaccination coverage and needs in developing countries [14]. Validity and reliability of the EPI method was previously assessed [15, 16]. A population parameter of 0.5 and design effect of 6 were used to allow for the multi-stage clustering sampling structure, resulting in a required sample size of 576 clusters (households). We hypothesized a 10% households with incomplete data thus aimed to select 11 households in every health area (57 in total), totaling 627 households.

The city of Yaoundé has 6 health districts, where each health district is composed of four to twelve health areas. Currently, there are a total of 57 health areas in Yaoundé. Each of these health areas has at least one community, called “quartier”. For the first stage of the sampling, one community was randomly selected from each of 57 health areas, resulting in a total of 57 communities. Then, from each of the randomly selected communities, 11 households were selected using convenient sampling method.

### Study protocol

In cooperation with the Faculty of Medicine and Biomedical Sciences of the University of Yaoundé I and a Nursing School, 20 surveyors were recruited and received a mandatory full day of training prior to the survey. The surveyors were informed on the methodology of the survey as well as the detailed procedures. Subsequently, a pilot study was conducted to evaluate the extent of surveyors’ understanding on the survey.

Using a structured survey form and variable dictionary, face-to-face interviews were conducted from 1 to 4 March, 2017. Twenty surveyors were divided into ten teams of two. Each day during the survey period, each team was assigned to cover one or two communities, where they conducted face-to-face interviews in at least 11 households per community. Before conducting surveys in the assigned community, surveyors visited the “Chefferie” of each community, for their approval to conduct community survey in the community. Upon receiving the Chefferie’s authorization, recruitment of participants was begun based on a door-to-door approach at residential properties. At the center of each sampled community, surveyors randomly selected a direction, and the most proximal house that lies in the chosen direction was selected to participate in the survey. From the first house, subsequent houses were selected by visiting the next available house. To track the households they visited and collect data on reasons for not participating into the survey, each survey team kept tracking log for every house they visited.

From each household selected, an adult over 21 years of age was selected to participate in the survey as a respondent. The selected participant from each household provided information on behalf of the entire family members. There was no particular method of selecting a respondent; any adult member of the family was able to participate regardless of his or her sociodemographic status. Only those who provided written consent were included in the study, and the survey took place in each selected household or other space where family’s privacy and confidentiality were ensured. No personally identifying information were collected through the surveys and participants were ensured that their answers were confidentially managed. Surveyors filled out the survey forms on behalf of the respondent. On completion, the respondents from each household were offered a gift (toothpaste or soap) for their time and participation. The survey continued until at least 11 households were surveyed in each community.

To ensure the quality of the data, all survey forms were collected at the end of each day and reviewed by the research team. Surveyors were immediately informed if there were any incompleteness or inaccuracy on the survey forms. On the following day, for the purpose of enhanced quality control, the research team conducted secondary quality control by randomly selecting one or two surveyed communities each day and revisiting the participated households to make sure the data were accurately collected. Surveyed data were subsequently entered into Microsoft Excel (Microsoft Corporation, Seattle, Washington, USA) by three independent data entry contractors.

### Data collection and measurements

The structured survey form that was used for the 2014 survey was used to collet quantitative data on demographic, socioeconomic, and healthcare usage in the previous year (Supporting information S1 File for French version and S2 File for English version). Demographic information included age, gender, number of family members, and address. Socioeconomic information included language, education, insurance, occupation, household income, and residential information (owned, rented, or other). For healthcare utilization, the number of outpatient visit, emergency room usage, and hospitalization in the past one year by each family member was collected.

The primary outcome measure of the study was healthcare utilization in Yaoundé before and after the establishment of CURY. The healthcare utilization outcomes of the current survey was compared with the outcomes of 2014 survey [6].

### Statistical analysis

Descriptive analysis was performed to compare the distribution of demographic and socioeconomic factors of the survey participants between the two survey years and statistical significance of the differences were analysed with Person’s χ^2^ test for categorical variables and Wilcoxon rank-sum test for continuous variables.

Proportions of the participants with outpatient and emergency unit usage, and hospitalization were calculated for both survey years and the statistical significance of the differences were evaluated using χ^2^ test.

Individual factors associated with healthcare utilization were examined using a multivariable logistic regression model. Sample weighting was carried out using 2017 Yaoundé population data from each health area. The base weight for each health area was calculated as the reciprocal of the final probability of selection for a participant from each health area.

P-values were calculated based on a two-sided significance level of 0.05. All statistical analyses were performed using SAS software V.9.4 (SAS Institute, Cary, North Carolina).

## Results

### Demographic findings of respondents

**Fig 2:**
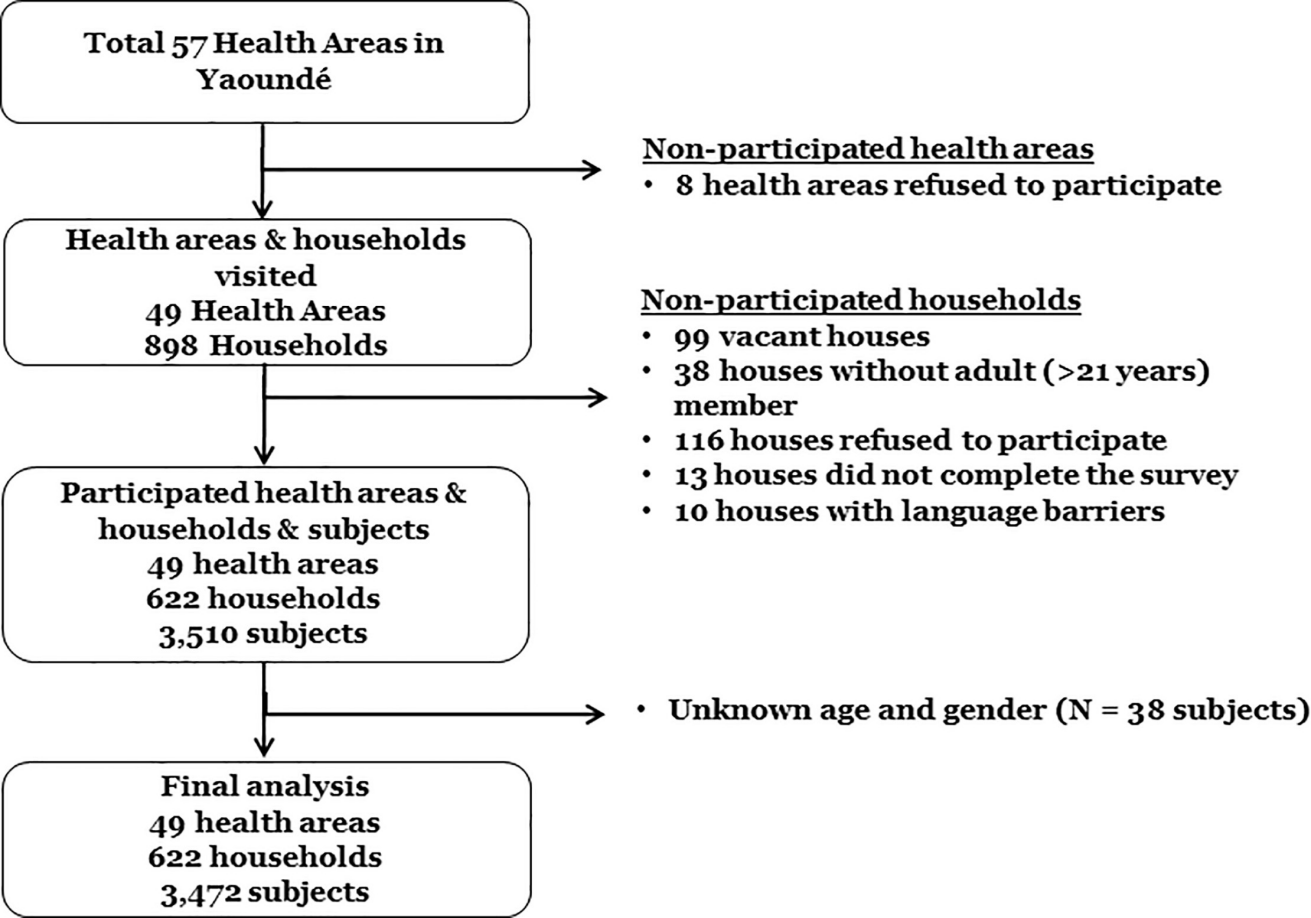
Flow diagram of the study participants in 2017 survey.

Among 898 households visited for the survey, 99 houses were vacant (11%). Of those answered the door, 38 houses only had non-adult (≤ 21 years old) members, 116 refused to participate, 13 houses did not complete the survey, and 10 houses had language barrier. A total 622 households (response rate 69.2%) with 3,501 subjects completed surveys. Excluding those with unknown age and gender, a total of 3,472 subjects from 622 households were included for the final analysis (Fig 2). In 2014 survey, a total of 619 households and 3,201 subjects were included for the final analysis.

Table 1 shows demographics of survey respondents by survey year. In both survey years, about 54% of the total respondents were female with majority of the respondents being in 0–17 (40.4% in 2014 and 43.2% in 2017) and 18–35 (38.3% in 2014 and 35.1% in 2017) age groups. Among 2017 survey respondents, 1,127 (32.4%) had household income of < XAF 25,000 (~USD 46) per week. Given the unknown/missing household income was 54.6% in 2017 survey, among the survey respondents with the known household income, 71.4% had household income of < XAF 25,000. In 2017 survey, those who responded as “unemployed” at the time of the survey was 12.8% among the total survey respondents. For both survey years, less than 3% of survey respondents had health insurance.

**Table 1:** Demographics of survey respondents by survey year.

	2014 Survey		2017 Survey		P-value*	
	N	Percent	N	Percent		
Total	3,201		3,472		
Gender					0.72
Female	1,720	53.7	1,881	54.2	
Male	1,481	46.3	1,591	45.8	
Age					<0.01
0–17	1,292	40.4	1,499	43.2	
18–35	1,227	38.3	1,220	35.1	
36–55	516	16.1	156	4.5	
56 and more	163	5.1	237	6.8	
Median (IQR)	22 (9–33)		21 (8–33)		0.19
Household income (XAF/week)					<0.01
1–10,000	481	15.0	428	12.3	
10,001–25,000	846	26.4	699	20.1	
25,001–50,000	474	14.8	329	9.5	
50,001 and more	98	3.1	122	3.5	
Unknown/Missing	1,302	40.7	1,894	54.6	
Occupation					<0.01
Non-physical labor	247	7.7	211	6.1	
Physical labor	413	12.9	582	16.7	
Unemployed	681	21.3	445	12.8	
Housewife	222	6.9	251	7.2	
Preschool/student/Other	1,303	40.7	1,903	54.9	
Unknown/missing	335	10.5	80	2.3	
Education					
Pre-primary schoolers	520	16.2	596	17.2	<0.01
Primary school	567	17.7	868	25.0	
Middle school	1,021	31.9	933	26.9	
High school	449	14.0	428	12.3	
University of higher	82	2.6	121	3.5	
Unknown/Missing	562	17.6	526	15.2	
Insurance					
Yes	86	2.7	104	3.0	<0.01
No	3,083	96.3	3,361	96.8	
Unknown/missing	32	1.0	7	0.2	
Language					
French	2,987	93.3	3,004	86.5	
English	107	3.3	97	2.8	
Other	93	2.9	370	10.7	
Unknown/missing	14	0.4	1	0.0	
Number of family members					<0.01
1–2	100	3.1	187	5.4	
3–6	1,687	52.7	1,763	50.8	
7–10	1,171	36.6	1,134	32.7	
11–14	135	4.2	225	6.5	
15 and more	108	3.4	163	4.7	
Residential information					<0.01
Owned	1,729	54.0	1,816	52.3	
Rented	1,199	37.5	1,627	46.9	
Other	18	0.6	17	0.5	
Unknown/missing	255	8.0	12	0.3	

Abbreviations: IQR = interquartile range; XAF = Central African CA Franc BEAC

*P-value based on Chi-square test for categorical variables and Wilcoxon rank-sum test for continuous variable.

### Changes in healthcare utilizations in Yaoundé from 2014 to 2017

Table 2 shows results of the healthcare utilization during the past one year for both 2014 and 2017 surveys. For 2017 survey, we were able to obtain the Yaoundé census data by health area thus both crude and weighted results were shown in2017. Overall, the weighted results for 2017 survey showed similar results compared with unweighted results.

**Table 2:** Healthcare utilization in the past 1 year by survey year.

	2014 Survey		2017 Survey*				
	No weighting (N = 3,201)		No weighting (N = 3,472)		With weighting (Weighted N = 2,997,204)		p-value**
	N	Percent	N	Percent	Weighted N	Percent (95% CI)	
*Number of Emergency Unit Usage*							0.08
Never	3,035	94.8	3,270	94.2	2,825,553	94.3 (93.3–95.2)	
Ever	155	4.8	202	5.8	171,651	5.7 (4.8–6.7)	
1 time	113	3.5	167	4.8	144,229	4.8 (3.9–5.7)	
2~3 times	30	0.9	27	0.8	21,521	0.7 (0.4–1.1)	
4~5 times	11	0.3	5	0.1	4,498	0.2 (0.0–0.3)	
6~7 times	1	0.0	1	0.0	443	0.0 (0.0–0.0)	
>7 times	0	0	2	0.1	960	0.0 (0.0–0.1)	
Unknown	11	0.3	0		0		
*Number of Outpatient Usage*							<0.001
Never	1,999	62.4	2,345	67.5	2,027,204	67.6 (65.7–69.6)	
Ever	1,175	36.7	1,127	32.5	970,000	32.4 (30.4–34.3)	
1 time	575	18.0	497	14.3	454,508	15.2 (13.7–16.7)	
2~3 times	361	11.3	351	10.1	302,041	10.1 (8.8–11.4)	
4~5 times	113	3.5	133	3.8	83,597	2.8 (2.2–3.4)	
6~7 times	44	1.4	86	2.5	71,700	2.4 (1.7–3.1)	
>7 times	82	2.6	60	1.7	58,154	1.9 (1.3–2.6)	
Unknown	27	0.8	0		0		
*Number of Hospitalization*							0.13
Never	2,882	90.0	3,154	90.8	2,737,208	91.3 (90.2–92.5)	
Ever	319	10.0	318	9.2	259996.0	8.7 (7.5–9.8)	
1 time	245	7.7	226	6.5	176,317	5.9 (4.9–6.8)	
2~3 times	48	1.5	74	2.1	66,203	2.2 (1.6–2.8)	
4~5 times	16	0.5	12	0.3	10,879	0.4 (0.1–0.6)	
6~7 times	2	0.1	4	0.1	4,527	0.2 (0.0–0.3)	
>7 times	1	0.0	2	0.1	2,069	0.1 (0.0–0.2)	
Unknown	7	0.2	0		0		

*Total population of Yaoundé in 2017 = 2,969,156

** P-value between 2014 survey and 2017 survey without weighting using Chi-square test

Among 3,472 respondents in 2017 survey, approximately 6% of the total respondents (5.8% in unweighted and 5.7% in weighted samples) in 2017 survey used the emergency unit during the past one year. During the 2014 survey, the emergency unit utilization was 4.8%, which was marginally significantly lower than the 2017 survey (p = 0.08). For outpatient usage, over 32% responded (32.5% in unweighted and 32.4% in weighted samples) having at least one time outpatient usage during the past one year in 2017, which was significantly lower than that of in 2014 survey (36.7%; p<0.001). For hospitalization, approximately 9% of the total survey respondents in 2017 (9.2% unweighted and 8.7% in weighted sample) responded that they were hospitalized in the past year, compared with 10.0% in 2014 survey participants (p = 0.13).

### Multivariable logistic analysis for emergency care utilization in 2017 survey

For 2017 survey respondents, being female (adjusted OR (aOR) = 1.79; 95% CI = 1.29–2.47) and language other than French or English (aOR = 1.53; 95% CI = 1.02–2.30) were associated with higher emergency care utilization (Table 3). On the other hands, younger age group (0–17 age group compared with 18–35 age group) and those with physical labor occupation (compared with unemployed) showed lower emergency care utilization in the past one year with aORs (95% CIs) of 0.57 (0.34–0.98) and 0.57 (0.35–0.92), respectively.

**Table 3:** Multivariable logistic analysis for emergency care utilization in the past 1 year, 2017 survey.

	Unadjusted OR	Adjusted OR
	(95% CI)	(95% CI)
Gender		
Male	1.00	1.00
Female	1.98 (1.46–2.69)	1.79 (1.29–2.47)
Age		
0–17	0.41 (0.29–0.58)	0.57 (0.34–0.98)
18–35	1.00	1.00
36–55	0.88 (0.59–1.31)	0.88 (0.58–1.34)
56 and more	1.02 (0.61–1.70)	1.02 (0.59–1.74)
Household income (XAF/week)		
1–10,000	1.00	1.00
10,001–25,000	1.36 (0.81–2.29)	1.32 (0.78–2.26)
25,001–50,000	1.45 (0.80–2.64)	1.63 (0.87–3.05)
50,001 and more	1.47 (0.66–3.28)	1.38 (0.59–3.19)
Unknown	1.02 (0.63–1.64)	1.04 (0.64–1.70)
Occupation		
Non-physical labor	0.65 (0.35–1.19)	0.72 (0.38–1.35)
Physical labor	0.52 (0.33–0.83)	0.57 (0.35–0.92)
Unemployed	1.00	1.00
Housewife	1.15 (0.71–1.87)	0.93 (0.55–1.56)
Preschool/student	0.32 (0.22–0.47)	0.43 (0.27–0.70)
Other/unknown/missing	0.73 (0.30–1.76)	0.81 (0.33–2.01)
Insurance Yes	1.00	1.00
No	1.23 (0.50–3.06)	1.33 (0.53–3.37)
Unknown/missing	NA	NA
Number of family members		
1–2	1.00	1.00
3–6	1.04 (0.53–2.03)	1.29 (0.65–2.56)
7–10	1.18 (0.60–2.34)	1.67 (0.83–3.39)
11–14	0.91 (0.38–2.19)	1.09 (0.44–2.69)
15 and more	1.41 (0.59–3.35)	1.56 (0.62–3.92)
Residential information		
Owned	1.00	1.00
Rented	1.00 (0.75–1.33)	1.21 (0.87–1.66)
Other/unknown/missing	0.58 (0.08–4.27)	0.67 (0.09–5.06)
Language		
French	1.00	1.00
English	0.36 (0.09–1.46)	0.35 (0.08–1.47)
Other/unknown/missing	1.66 (1.12–2.45)	1.53 (1.02–2.30)

Abbreviations: OR = odds ratio; CI = confidence interval; XAF = Central African CA Franc BEAC

NA = not applicable

## Discussion

To our knowledge, this is the first before-and-after survey assessing the changes of emergency care utilization after the establishment of an emergency center in Africa in 2015 (the CURY). We observed a one percent increase in emergency care utilization between 2014 and 2017 in Yaoundé, Cameroon, using community-based surveys, which provide comprehensive population-based estimation of healthcare utilization of residents of Yaoundé. While the increase was statistically marginally significant, this 1% increase in emergency care utilization may be extrapolated to about 32,000 people in the 3.2 million Yaoundé population. Given that there were 6 tertiary care centres in Yaoundé during 2016 and 2017, including the CURY, this increase can be further implied to over 5,300 additional residents seeking emergency care per facility.

While an emergency care system is a crucial component of the national health systems, particularly in LMIC, the development of emergency care has not been a priority in resource-limiting settings [17]. As Sub-Saharan Africa is experiencing a rapid epidemiological transition with the growing burden of non-communicable diseases, acute medical and surgical needs have been also growing significantly. However, providing effective emergency care has been severely lacking in LMIC, mainly due to limited infrastructure, supplies, and properly trained human resources to deliver quality emergency care [18]. The current health situation of Cameroon is also characterized by a significant increase of non-communicable diseases. According to the WHO, estimated annual injury-related mortality and disability-adjusted life-years (DALY) in Cameroon were 101.8 and 4,430 DALY per 100,000 population, respectively, putting the total contribution of burden of disease due to injury roughly on par with that of malaria [11]. Moreover, the latest WHO data reports that stroke and ischemic heart disease deaths are among the top 10 causes of deaths in Cameroon [19]. Therefore, a significant burden of diseases observed in Cameroon is attributable to time-sensitive illnesses and injuries.

In 2014, we conducted a community-based survey to assess the burden of emergency illnesses and the demands and usage of emergency care by Yaoundé residents within the past 1 year [6]. In the survey, 34.8% of the total respondents experienced emergency conditions in the past year but only 7.3% of them used emergency units and 68.8% of them reported unmet needs for emergency care, demonstrating the high incidence of emergency conditions, low usage and high unmet needs for emergency care in Yaoundé, Cameroon. Despite its urgent need of emergency care system in Cameroon, total public expenditure on health in Cameroon sharply declined from 2010 (5.3% of the GDP) to 2011 (4.0%), and has remained around 4% of the total GDP over the past several years [10]. Furthermore, growing numbers of road traffic accidents and inadequate prehospital systems, coupled with the limited number of trained professionals and appropriate equipment, there has been a continuous rise in the number of deaths from emergency situations.

The CURY is a special category 2 public hospital, which is specialized in the management of medical and surgical emergencies. The CURY missions are: (1) The management of medical and surgical emergencies; (2) The management of extra-hospital emergencies; (3) Regulation, coordination and mobilization of resources and capacities of facilities in emergency and disaster situations; (4) Operational participation in emergency plans in collaboration with other partners in charge of civil protection; and (5) Participation in the training of medical and paramedical personnel. Since its opening, the CURY has admitted more than 7,000 emergency patients each year.

In February 2017, approximately 20 months after the opening of the CURY, we conducted an after survey to assess change of healthcare utilization in Yaoundé. Compared with 2014 survey, we observed a marginal increase in the emergency care utilization in 2017, while both outpatient usage and hospitalization were decreased from 2014 to 2017. Although we cannot determine that the increase of emergency care utilization is solely due to the establishment of the CURY, we still believe that the establishment of this new emergency center has a great impact on the communities of Yaoundé by serving emergent patients. Nevertheless, current rate of emergency care utilization in Yaoundé is still exceedingly low than those of high income countries or even in other African countries [20].

Our study has several strengths. First, this study assessed emergency care utilization of the Yaoundé residents. Due to its unpredictable and time-sensitive nature, emergency-related data are often difficult to capture [21]. Particularly, data on emergency care utilization are sparse [22]. Therefore this study provides insights into emergency care utilization rate in Yaoundé, Cameroon. Second, our sequential surveys allow us to assess the changes of healthcare utilization of Yaoundé residents over time. For both surveys, we followed the same protocols and sampling methods to make the results comparable. Another strength of this study is representativeness of our sample. While we did not have Yaoundé population data to weight our data in 2014 survey, we were able to obtain population data in 2017. The weighted data was very similar to unweighted data, demonstrating that our samples are good representative of the Yaoundé population.

There are several limitations in this study. First, for each survey (2014 and 2017 surveys), we had different groups of surveyors conducting the surveys therefore there is a possibility of misclassification. Although all the surveyors in both surveys had to attend a full-day of mandatory training session, trained with same study protocol, and data quality control protocol were put in place, there still might have misclassification between surveyors of two different survey years. Second, one adult survey responder from each household answered for all household members for the previous year’s emergency condition. The average family size of the surveyed households were 6.7 with maximum number of family members being 20. Therefore, there is a possibility of recall bias to accurately remember each family member’s healthcare utilization in the past 1 year. Third, the appropriate use of emergency centres was not assessed in the survey. Therefore, we cannot be sure whether the 1% increase in the emergency care utilization we observed in this study represents the true increase in the emergency care utilization by emergent patients or not. Last, because we were not able to collect data on the names of the healthcare facilities participants visited in the past 1 year, we cannot be sure that the significant increase of the emergency care utilization from 2014 to 2017 was due to the establishment of the CURY. However, between two survey periods, there were no newly opened emergency center, tertiary hospitals or teaching hospitals in Yaoundé beside the CURY.

## Conclusions

This study provides evidence that the emergency care utilization in Yaoundé, Cameroon has increased from 2014 to 2017, after the establishment of the CURY. Although we believe that the establishment of the CURY has attributed to the increase in the utilization of emergency care in Yaoundé, it only provided hospital-level care to patients in need of emergency care. Emergency medical services (EMS) system is multi-factorial system, which involves from pre-hospital to hospital level. Therefore development of an integrated EMS system should be a priority within global health agenda, particularly in limited-resources settings. Moreover, comprehensive assessment and continuous monitoring of healthcare utilization in Cameroon, particularly emergency care, would help development of an evidence-based, effective emergency care system and health policies in Cameroon.

## Supporting information

S1 File Survey Questionnaires in Original Version (French). (PDF)

S2 File Survey Questionnaires in English Version. (PDF)
