# Supplementary material for: Changes in the healthcare utilization after establishment of emergency centre in Yaoundé, Cameroon: A before and after cross-sectional survey analysis
Source: PLoS One. 2019 Feb 8;14(2):e0211777. doi: 10.1371/journal.pone.0211777 (PMC6368305; doi:10.1371/journal.pone.0211777)
Supplement: S1 File — (PDF) [file pone.0211777.s001.pdf]

S1 File. Survey Questionnaires in Original Version (French)

1. Information du Participant

Nom		Genre	◻ Masculin ◻ Féminin	Age	
Nombre de membres de famille*		**membre de la famille est celui qui a vécu dans la résidence pendant la période d'1an passée (les membres décédés sont inclus)*			
No. d'enquête	District ( ) Maison ( )	No. du Membre	Membre ( )		
Adresse de résidence	(si l'adresse n'est pas disponible, indiquer sur la carte)				
Adresse du centre de sante de district	(si l'adresse n'est pas disponible, indiquer sur la carte)				

2. Information Socioéconomique

Langue de naissance	◻ Anglais ◻ Français ◻ Autres	Education	( ) ans	Assurance	◻ Oui ◻ Non
Travaille	◻ Non-Manuel ◻ Manuel (régulier) ◻ Manuel (non régulier) ◻ Sans emploi ◻ Ménagère ◻ Autres ◻ Préfère ne pas dire ◻ Inconnu				
Revenu des ménages	( ) XAF/semaine	Information résidentiel	◻ Vous appartiens ◻ En location ◻ Autre		

3. Utilisation des soins de santé dans l'année écoulée

	Nombre	Le motif le plus fréquent (symptôme) [1]	Satisfaction [2]
Consultation externe			
Salle d'urgence			
Hospitalisation			

4. Besoins non satisfaits pour des symptômes ou blessures graves et urgents dans l'année écoulée

Symptômes d'urgence	Définition	Nombre d'événements	Nombre de non-visites	Si non visite, quel était la principale raison?
Neurologique	Trouble aiguë de la conscience, l'anomalie neurologique aiguë, vertiges, trauma à la tête (accompagné de vomissements, perte de conscience)			
Cardiovasculaire	Arrêt cardiaque, détresse respiratoire aiguë, douleur thoracique, palpitations, pouls irréguliers et choc, hyperventilation			
Intoxication / trouble métabolique	Déshydratation sévère, usage de drogues, intoxication, trouble métabolique aiguë (insuffisance hépatique, insuffisance rénale, etc.)			
Urgence chirurgicale	Abdomen aigu, brûlures graves (> 18% de la surface totale du corps), plaie pénétrante, fracture ouverte, désarticulation, lésion vasculaire, traumatismes multiples, traumatisme nécessitant une chirurgie d'urgence, troubles de la miction			
Hémorragie	Hémoptysie, hémorragie sans hémostase, une hémorragie gastro-intestinale aiguë			
Urgence Pédiatrique	Trouble convulsivant pédiatrique, hyperthermie supérieure à 38°C (8 ans ou moins)			
Urgence obstétrique	Parturition, violence sexuelle			
Urgence Psychiatrique	Trouble psychiatrique qui menace soi-même ou d'autres			

Allergies	Réaction allergique avec œdème faciale			
Urgence Ophtalmologique	Lésions oculaires due à des produits chimiques, dommages aiguës de la vision			
Corps étranger	Corps étranger des oreilles, des yeux, ou de l'anus			
Trauma	Blessure, lacération, fracture, etc.			

5. Décès de membres de la famille dans l’année écoulée

Décès de membres de la famille	□ Oui (Répondre aux questions suivantes) □ Non

Lle nom du défunt	Âge au décès	Symptôme [1]	Acuité [4]	A visité hôpital?	Lieu du décès	Diagnostic
				□ Non □ Centre de santé □ Salle d'urgence □ Hôpital	□ Maison □ Centre de santé □ Salle d'urgence □ Hôpital	
				□ Non □ Centre de santé □ Salle d'urgence □ Hôpital	□ Maison □ Centre de santé □ Salle d'urgence □ Hôpital	
				□ Non □ Centre de santé □ Salle d'urgence □ Hôpital	□ Maison □ Centre de santé □ Salle d'urgence □ Hôpital	

Noter:

1. Pour les raisons les plus fréquentes de l'utilisation des soins de santé (symptômes), utilisez la catégorie: 4. Besoins non satisfaits pour des symptômes sévères et urgents ou des traumas dans l’année écoulée.
2. Evaluer Le niveau de satisfaction comme il suit: 1) très insatisfaisant; 2) insatisfaisante; 3) moyen; 4) satisfaisant; 5) très satisfaisante.
3. Pour les raisons de ne pas visiter l'hôpital, enregistrer le motif principal comme il suit: 1) l'accessibilité - longue distance à l'hôpital; 2) l'accessibilité - le manque de transport; 3) l'accessibilité - le manque d'hôpital; 4) le cout - question économique; 5) la désapprobation sociale et familiale – conseils de la famille et/ou d'un ami; 6) autre (description).
4. Evaluer le niveau d'acuité comme il suit: 1) une maladie aiguë (accident par exemple); 2) des symptômes aigus de maladies chroniques; 3) autre ; 4) inconnu.
