# Supplementary material for: Changes in the healthcare utilization after establishment of emergency centre in Yaoundé, Cameroon: A before and after cross-sectional survey analysis
Source: PLoS One. 2019 Feb 8;14(2):e0211777. doi: 10.1371/journal.pone.0211777 (PMC6368305; doi:10.1371/journal.pone.0211777)
Supplement: S2 File — (PDF) [file pone.0211777.s002.pdf]

S2 File. Survey Questionnaires in English Version

**1. Participant Information**					
Name		Gender	☐ Male ☐ Female	Age	
Number of family members*		**Family members are defined as those who had lived together in the residential address for the past 1 year (include deceased members)*			
Survey No.	District ( ) Household ( )	Member No.	Member ( )		
Residential address	(If address is not available, indicate on a map.)				
District Health Center address	(If address is not available, indicate on a map.)				

**2. Socioeconomic information**					
Native language	☐ English ☐ French ☐ Other	Education	( ) year	Insurance	☐ Yes ☐ No
Occupation	☐ Non-physical labor ☐ Physical labor (regular) ☐ Physical labor(casual) ☐ Unemployed ☐ Housewife ☐ Other ☐ Prefer not to say ☐ Unknown				
Household Income	( ) XAF/week	Residential information	☐ Owned ☐ Rented ☐ Other		

**3. Healthcare utilization in the past 1 year**			
	Number	Most frequent reason (symptom) [1]	Satisfaction [2]
Outpatient visit			
Emergency room			
Hospitalization			

4. Unmet needs for severe and emergency symptoms or injuries in the past 1 year

Emergency symptoms	Definition	Number of events	Number of non-visits	If not visited, what was the primary reason? [3]
neurologic	Acute abnormal consciousness, acute neurologic abnormality, vertigo, head injury (accompanied with vomiting, unconsciousness)			
Cardiovascular	Cardiac arrest, acute respiratory distress, chest pain, palpitation, irregular pulsation and shock, hyperventilation			
Intoxication/metabolic disorder	Severe dehydration, drug use, intoxication, acute metabolic disorder (hepatic failure, renal failure, etc.)			
Surgical emergency	Acute abdomen, severe burns (>18% of total body surface), penetrating injury, open fracture, disarticulation, vascular injury, multiple trauma, trauma requiring surgical emergency, micturition disorder			
Hemorrhage	Haemoptysis, hemorrhage with no hemostasis, acute gastrointestinal bleeding			
Pediatric emergency	Pediatric convulsive disorder, hyperthermia above 38°C (8 years old or younger)			
Obstetric emergency	Parturition, sexual violence			
Psychiatric emergency	Psychiatric disorder that threatens oneself or others			
Allergies	Allergic reaction with facial edema			
Ophthalmology emergency	Eye injury due to chemicals, acute eye vision damage			
Foreign body	Foreign body of ears, eyes, or anus			
Trauma	Wound, laceration, fracture, etc.			

5. Death of family members in the past 1 year						
Death of family members		□ Yes (answer the following) □ No				
Deceased name	Age/sex at death	Symptom [1]	Acuity [4]	Visited hospital?	Location at death	Diagnosis
				□ No □ Health center □ Emergency room □ Hospital	□ Home □ Health center □ Emergency room □ Hospital □ Other	
				□ No □ Health center □ Emergency room □ Hospital	□ Home □ Health center □ Emergency room □ Hospital □ Other	
				□ No □ Health center □ Emergency room □ Hospital	□ Home □ Health center □ Emergency room □ Hospital □ Other	

Note:

1. For most frequent reasons of healthcare utilization (symptom), use the category in: 4. Unmet needs for severe and emergency symptoms or injuries in the past 1 year.
2. Rate the level of Satisfaction as one of the following: 1) very unsatisfactory; 2) unsatisfactory; 3) average; 4) satisfactory; 5) very satisfactory.
3. For reasons of not visiting hospital, record the primary reason as one of the following: 1) accessibility – long distance to hospital; 2) accessibility – lack of transportation; 3) accessibility – lack of hospital; 4) affordability - economic issue; 5) social and familial disapproval – family and/or friend's advice; 6) other (description).
4. Rate the level of acuity as one of the following: 1) acute illness (e.g. accident); 2) acute symptoms of chronic disease; 3) other.
